# Intelligent Screening Systems for Cervical Cancer

**DOI:** 10.1155/2014/810368

**Published:** 2014-05-11

**Authors:** Yessi Jusman, Siew Cheok Ng, Noor Azuan Abu Osman

**Affiliations:** Department of Biomedical Engineering, Faculty of Engineering Building, University of Malaya, 50603 Kuala Lumpur, Malaysia

## Abstract

Advent of medical image digitalization leads to image processing and computer-aided diagnosis systems in numerous clinical applications. These technologies could be used to automatically diagnose patient or serve as second opinion to pathologists. This paper briefly reviews cervical screening techniques, advantages, and disadvantages. The digital data of the screening techniques are used as data for the computer screening system as replaced in the expert analysis. Four stages of the computer system are enhancement, features extraction, feature selection, and classification reviewed in detail. The computer system based on cytology data and electromagnetic spectra data achieved better accuracy than other data.

## 1. Introduction

Cervical cancer is a leading cause of mortality and morbidity, which comprises approximately 12% of all cancers in women worldwide according to World Health Organization (WHO). In fact, the annual global statistics of WHO estimated 470 600 new cases and 233 400 deaths from cervical cancer around the year 2000. As reported in National Cervical Cancer Coalition (NCCC) in 2010, cervical cancer is a cancer of the cervix which is commonly caused by a virus named Human Papillomavirus (HPV) [1]. The virus can damage cells in the cervix, namely, squamous cells and glandular cells that may develop into squamous cell carcinoma (cancer of the squamous cells) and adenocarcinoma (cancer of the glandular cells), respectively. Squamous cell carcinoma can be thought of as similar to skin cancer because it begins on the surface of the ectocervix. Adenocarcinoma begins further inside the uterus, in the mucus-producing gland cells of the endocervix [2].

Cervical cancer develops from normal to precancerous cells (dysplasia) over a period of two to three decades [3]. Even though the dysplasia cells look like cancer cells, they are not malignant cells. These cells are known as cervical intraepithelial neoplasia (CIN) which is usually of low grade, and they only affect the surface of the cervical tissue. The majority will regress back to normal spontaneously. Over time, a small proportion will continue to develop into cancer.

Based on WHO system, the level of CIN growth can be divided into grades 1, 2, and 3. It should be noted that at least two-thirds of the CIN 1 lesions, half of the CIN 2 lesions, and one-third of the CIN 3 lesions will regress back to normal [3]. The median ages of patients with these different precursor grades are 25, 29, and 34 years, respectively. Ultimately, a small proportion will develop into infiltrating cancer, usually from the age of 45 years onwards.

In 1994, the Bethesda system was introduced to simplify the WHO system. This system divided all cervical epithelial precursor lesions into two groups: the Low-grade Squamous Intraepithelial Lesion (LSIL) and High-grade Squamous Intraepithelial Lesion (HSIL). The LSIL corresponds to CIN1, while the HSIL includes CIN2 and CIN3 [4].

Since a period of two to three decades is needed for cervical cancer to reach an invasive state, the incidence and mortality related to this disease can be significantly reduced through early detection and proper treatment. Realizing this fact, a variety of screening tests have therefore been developed in attempting to be implemented as early cervical precancerous screening tools.

## 2. Methodology

This paper reviews 103 journal papers. The papers are obtained electronically through 2 major scientific databases: Google Scholar (http://scholar.google.com.my/) and Scopus (http://www.scopus.com/home.url). In the databases, the IEEE and Science Direct databases will be included already. Since there are various aspects being reviewed here, four sets of keywords have been used. The first set contains Cervical Cancer, Feature Extraction, and Intelligent System, which give an overview of an intelligent system for cervical cancer detection. The second set contains Cervical Cancer, Image Processing, and Intelligent System. The third set is made up of Cervical Cancer, Image Processing, and Classification. The final set contains Cervical Cancer, Features Extraction, and Image Processing.

In order to ensure a quality review, the academic papers reviewed here are limited to peer reviewed journal papers. Recent conference papers published in the year 2010 onwards are also considered as the work is up to date and the journal related to this work has yet to be published. However, certain conference papers that showed excellent results or used methods that are currently unpopular are also included to give a more complete perspective of the work done in this field.

## 3. Screening for Cervical Carcinoma

**Figure 1:**
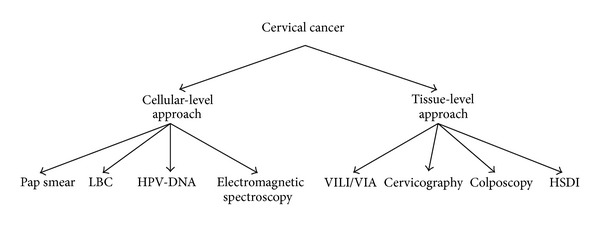
Taxonomy of cervical cancer screening.

Screening programs for cervical cancer have been implemented in developing countries for decades and have shown to be effective in reducing the overall mortality from this disease. There are two main diagnostic screening approaches for cervical cancer as presented in Figure 1:

1. diagnostic screening approach based on cellular level (i.e., Pap smear, liquid based cytology (LBC), HPV-DNA testing, and electromagnetic spectroscopies);
2. diagnostic screening approach based on the tissue level (i.e., visual inspection after applying Lugol's iodine (VILI) or acetic acid (VIA), cervicography, colposcopy, and hyperspectral diagnostic imaging (HSDI)).

For diagnostic screening based on cellular-level, the specimen collections are required before it is analyzed for the expert analysis results. In contrast, specimen collection is not required for diagnostic screening based on tissue-level. The expert analysis is required for cervix images visually after applying certain liquid into the cervix surface. Detail of standard procedure, advantages, and disadvantages for Pap smear, LBC, HPV-DNA, VILI/VIA, cervicography, and colposcopy techniques can be found in [5].

On the other hand, current technologies have investigated the cervical cell from the specimen under the spectroscopy equipment inducing an electromagnetic light. There are several techniques utilized for cervical cancer detection:

1. image results: fluorescent in situ hybridization (FISH) [6–11];
2. spectra results: Raman spectroscopy [12, 13], fluorescence spectroscopy [14, 15], and Fourier transform infrared (FTIR) spectroscopy [16–24].

On the other hand, there is an alternative technique based on tissue level known as hyperspectral diagnostic imaging (HSDI). The surface of the cervix is scanned with ultraviolet and white light for detecting lesions [25–27]. The scanning is achieved one line at a time, with the scan time varying from 12 to 24 seconds. By taking a series of scan lines, a hyperspectral data cube is obtained. This hyperspectral data cube contains spatial information (pixels) in two dimensions and spectral information (bands) in the third dimension [27]. This technique produces a 3D cervix image that is easier to interpret.

Based on the references, the techniques have several features required for considerations as summarized in Table 1. Each of the technique has advantages and disadvantages individually. Almost all of the techniques have on average nonexpensive or low cost features [5, 28, 29]. However, the EMS machines as well as microscope (for Pap smear and/or ThinPrep) and high resolution camera (for colposcopy and/or HSDI) are quite expensive to be bought for the beginning proses as screening technique but it is cost effective in the long run as no analysis from pathologist is required.

For the cellular-level techniques, the specimen collections require certain duration time and the results cannot be obtained spontaneously after specimen collection process due to need of the next process for the expert reading (i.e., image, spectrum, genetic material, etc.). As for the tissue-level, analysis of the experts could be obtained after reading the images captured by the camera.

Based on Table 1, the HPV-DNA is not subjective due to the genetic material for chemistry analysis on the cell. Similarly, the EMS techniques are also not subjective. They have quantitative results used for analysis. However, the HPV-DNA and the VILI/VIA techniques are not possible to interface in real time so they cannot be developed into an intelligent system. Therefore, intelligent systems for cervical precancerous is limited to the six possible techniques in real time as presented in Table 1.

## 4. Intelligent System Approach to Cervical Cancer

**Figure 2:**
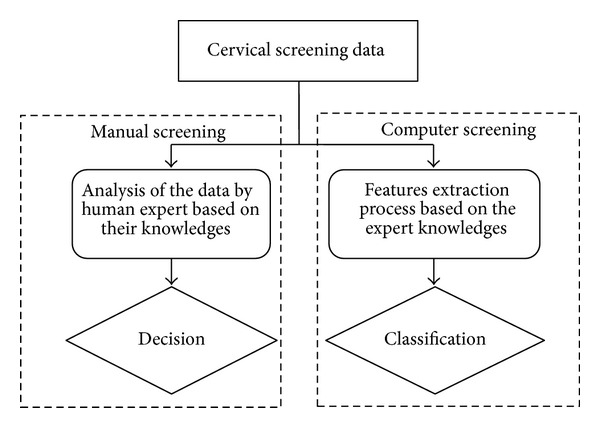
Comparison of analysis screening system by human expert and machine.

The cervical screening methods mentioned in Section 3 are highly dependent on the skill of the experts. However, their judgment may be subjective and often leads to considerable variability [5]. Aside from that, the limited number of experts and the large number of patients resulted in a long queue for the screening process. To overcome these problems, computational tools have been developed for automated cancer diagnosis as drawn in Figure 2. The automated cancer diagnosis facilitates objective judgment complementary to expert's decision.

Figure 2 shows the principle comparison of the computer screening technique and the human expert. The feature's extraction and classification by the computer replace the analysis and decision of human experts. Currently, the requirement for analysis based on computer screening increases. A number of researches were carried out specifically with the attempts to automate the classification [30, 31]. The results of several research to indicate that computer-imaging-assisted screening significantly increases the detection of cervical abnormalities compared to the manual screening [32, 33]. Consequently, automated screening devices would be a tremendous improvement for reducing the likelihood of human errors.

**Figure 3:**
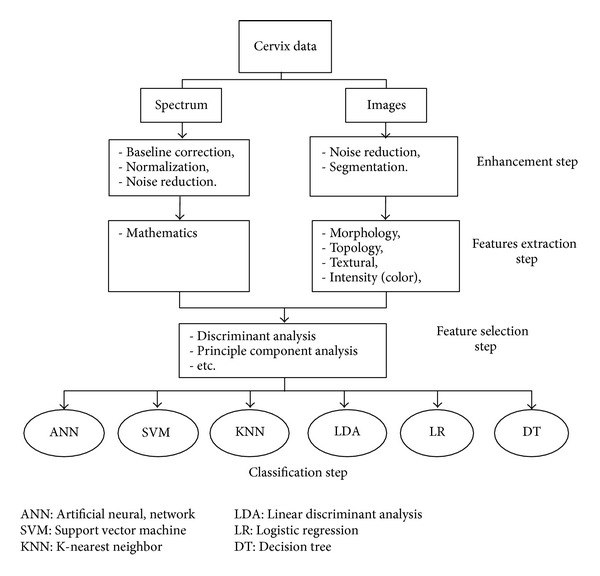
Intelligent cervical cancer classification systems.

A typical computer screening system involves four stages, namely, data enhancement, features extraction, features selection, and classification as shown in Figure 3. Aside from visual inspection after applying Lugol's iodine (VILI) or acetic acid (VIA) and HPV-DNA Testing, the data from the other screening techniques can be digitalized and fed into the intelligent computer screening system. These data can be categorized as images or spectra.

In the enhancement stage, the image or spectra will be processed in order to eliminate the noise to increase the signal to noise ratio. For images, this stage also involves determination of the region of interest to be segmented out for further processing. For the images, features are extracted either at the cellular or at the tissue-level. Basically, the morphology, texture, shape, and/or intensity of the cell/tissue image are extracted as features. For spectra, the features are height of intensity, shift of wave number, and corrected area and area under peaks of the spectra.

The main purpose of feature's selection is to reduce the number of features used in classification while maintaining acceptable classification accuracy. Feature's selection includes methods such as sequential backward selection [34], sequential forward selection [35], sequential floating search method [36], discriminant analysis [37], and principal component analysis [17].

After features selection step, several classifiers can be employed to obtain classification performance based on the used features. Different classification results can be performed by the different features used [38]. The aim of diagnosis step is to distinguish benignity and malignancy or to classify different malignancy levels by making use of extracted features. This step uses statistical analysis of the features and machine learning algorithms to reach a decision. An overview of these four stages is given in Figure 3. In the following sections, we will study each of these steps in detail.

Nowadays, there are several instruments which have been used to screen for abnormal cervical cells such as semiautomated or interactive system (PAPNET) and automated systems (AutoPap 300, FocalPoint, and ThinPrep Imaging System (TIS)) [30, 33, 39–41]. These instruments have been approved by United States Food and Drug Administration (USFDA) for screening system. These instruments utilize algorithmic image analysis to extract morphological features. Most of these systems help the expert to perform better diagnosis by improving cervical cell images quality so that the morphological features can be seen easily.  Table 2 summarizes the instruments to view their advantages and disadvantages.

In fact, to build the current intelligent cervical screening system, two types of raw data (i.e., digital images and spectra) as presented in Section 3 can be used for the purposes. To construct the intelligent system, data enhancement (optional), features extraction, and classification steps are applied to the raw data to obtain good screening results approach of the human expert knowledge in some areas of their expertise [42, 43]. Therefore, here we review some current features extraction techniques and classification of two types of cervical data.

### 4.1. Data Enhancement

**Figure 4:**
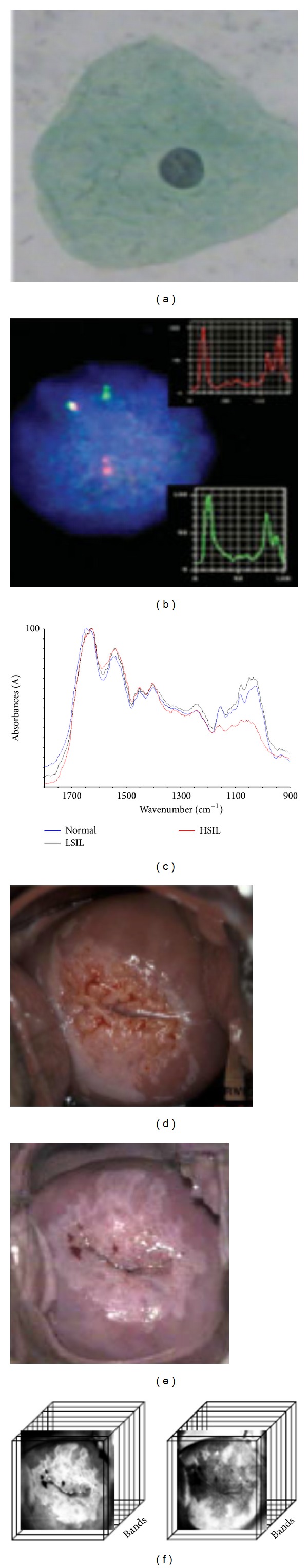
Cervical data used for intelligent classification. Celluler-level features; (a) cytology image, (b) FISH image, and (c) optical spectra. Tissue-level features; (d) cervicography, (e) colposcopy, and (f) optical image (HSDI).

As stated in earlier section, there are two types of cervical cancer data, which are spectrum and image as presented in Figure 4. The main aim of the enhancement stage is to reduce noise and for the image data to determine the area of interest as well. Due to a considerable amount of noise that arises from the staining process, it is usually necessary to reduce the noise prior to the segmentation process. In some studies, noise reduction and segmentation are carried out at the same time.

The aim of noise reduction for the spectrum is to reduce high frequency noise contained in the spectrum that can be from either noise conducted through power lines or radiated through the hot air in the electromagnetic spectroscopy equipment [44]. Savitzky-Golay (SG) filter is currently being used widely for smoothing the spectroscopy spectra [45–52]. The SG filter has boundary problems which can be solved by using other techniques such as Binomial and Chebyshev filters [53–55].

For image data, image noise is random (not present in the object image) variation of brightness or color information in images and is usually an aspect of electronic noise. The noise is an undesirable by-product of image capture that adds spurious and extraneous information. It can compromise the level of detail in cervix image, and so reducing this noise can greatly enhance the image. There are several noise reduction techniques offered by many researchers for the automated cervical cancerous applications system as follows.

- Based on pixel intensity: thresholding [43, 56–60] and filtering techniques [57, 60, 61].
- Based on shape: mathematical morphology [58, 60, 62].
- Based on the gradient: [63, 64].

Thresholding and filtering are to reduce the noise by making use of the pixel intensities. In threshold, the intensity histogram of an image is employed to determine the threshold value where the pixels are considered to be noise. For example, the Otsu method determines an optimal threshold which minimizes the within-class variance [62]. This method yields satisfactory results when the numbers of pixels in each class are close to each other. One weakness of threshold is that all pixels under the threshold value can be noise even the pixel information which is important. Conversely, the pixels over the threshold value can be information even the pixels which are noise. In filtering, the value of a pixel is transformed to a new value which is computed as a function of the values of pixels located in a selected neighborhood around this particular pixel. This is an improvement over the threshold method.

Another method for noise reduction which reduces the noise based shape characteristics of the input image is to use mathematical morphology. The basic morphological operators are the erosion and dilation of the set with a structuring element. These two basic transformations give two other transformations known as opening and closing. Opening is the erosion of an image followed by the dilation; it breaks narrow isthmuses and eliminates small objects and sharp peaks in the image. On the other hand, closing is the dilation of an image followed by the erosion; it fuses narrow breaks and fills tiny holes and gaps in the image [58, 65]. This technique can enhance region of interest (ROI) of the images perfectly by removing and adding small shape in the focused images.

Meanwhile, the segmentation process is used to detect the region of interest in the cervical image. The process is a key procedure in automating computer-aided diagnostic systems, because accurate images segmentation could help to reduce the processing time and increase the sensitivity rates. The segmentation method should be chosen depending on the type of the features to be extracted. Several segmentation techniques have been proposed and applied in cervix images as follows.

 (i) Based on shape: [57, 58, 66].
 (ii) Based on color: [61, 67–70].
(iii) Based on texture: [61, 71].
(iv) Based on contour: [59, 72–74].

### 4.2. Features Extraction

Automated cervical cancer diagnosis relies on using the information obtained from (i) the abnormalities in the cell structures (*cellular-level*) and (ii) the abnormalities in the cell distribution across the tissue (*tissue-level*). Many researchers have applied various captured techniques for the automated classification of cervical cancer. The techniques are cytology, FISH, and electromagnetic scanner for cellular level while cervicography, colposcopy, and HSDI are used for tissue level. Features are then extracted from data of the techniques as presented in Table 3.

The features are extracted to quantify these changes in a given tissue. In order to measure the abnormalities at the cellular/tissue level, size and shape, ratio, topology, texture, and color intensity can be used as features listed in Table 3. The features are extracted and represented by a value to be used in the intelligent system.

#### 4.2.1. Size and Shape Feature

A cell includes a nucleus surrounded by cytoplasm. As a traditional way, a pathologist evaluates the cytoplasm and the background of slide. The abnormality features are described as size (i.e., there is an increased size of the nucleus compared to the cytoplasm), shape (i.e., smooth, circular, and oval outline belongs to a normal nucleus), texture (i.e., rough textures belong to an abnormal nucleus), chromaticity (i.e., abnormal nucleuses are darker than normal ones) [62]. The quantification of these properties enables differentiating the malignant cells from those of benign and normal.

The size is expressed by the radius, area, and perimeter of the cell. Suppose that *S* = {*s*_1_, …, *s*_*n*_} is a set of the boundary points of a segmented cell/nucleus and *C* is the centroid of these boundary points, a sample of a nucleus with its boundary points. On the other hand, the shape is expressed by the length of the major and minor axes, symmetry, and circularity. The size and shape features defined on the set of the boundary points, *S*, are given as follows.

(i)  Radius *r* is defined as the average length of the radial lines towards every boundary point. Mathematically,
  (1) $$\begin{array}{c} r=\frac{\sum_{i=1}^{n}\left|s_{i}C\right|}{n}. \end{array}$$
(ii)  Area is the number of pixels within the boundary.
(iii) Perimeter *P* is measured as the sum of the distances between every consecutive boundary point. Mathematically,
  (2) $$\begin{array}{c} P=\left|s_{n}s_{1}\right|+\overset{n-1}{\underset{i=1}{\sum }}\left|s_{i}s_{i+1}\right|. \end{array}$$
 (iv) Major axis is the longest chord that goes through the center and minor axis is the line that is perpendicular to the major axis and that goes through the center.
(v) Circularity is quantified by drawing chords between nonadjacent boundary points and checking whether or not the boundary points lie inside these chords.

Several researchers have identified capability of the size and shape features to classify the cervix using the cytology image [72, 73, 75], FISH image [60, 76, 77], and electromagnetic spectrum [24, 78]. Besides these features, the ratio of the same feature for different parts of a biological structure is used as another feature. For example, the nuclear area/cytoplasm area ratio [73] and the corrected area under peak A/under peak B ratio [78] are such a kind of features.

From cytology images as presented in Figure 4(a), the specific features as listed in Table 3 (i.e., size, shape, and ratio), namely, average nucleus size [72, 73, 75], average cytoplasm size [75], average cell size [72], cytoplasm circularity [75], nucleus circularity [24, 75], percentage of cell coverage [72], ratio of a nucleus to cytoplasm size [72, 75], and percentage of empty cells [72], are partially used to be an input attribute to the classification system.

For FISH image, the features from labeled biomarker spots of chromosomes 3 (red spot) and X (green spot) are the size of each colored spot [60, 76, 77], the effective radius of each red or green spot computed as the radius of a circle that had the same size as the colored spot [60, 76, 77], and the circularity of each colored spot [60, 77].

Meanwhile, from the electromagnetic spectra, the features are shift of peak frequency [24], absorbance value, and area under the spectra. For the absorbance features, the corrected absorbance value and ratio of the absorbance/corrected absorbance values for certain regions in a spectrum are derived from the features [24, 78]. Then, from area under the spectra, the features can be taken as corrected area and ratio of the area/corrected area values for certain regions in one spectrum [78].

At the case of tissue-level image, the shape feature is applied to differentiate the cervix images. The anatomical region features of the cervix (as marked by the medical experts) can be characterized by their elliptical or circular shapes; hence, the ellipse and the circle are chosen for the shape models. A vast amount of work was done to embed prior-shape information into a segmentation task. A popular approach is to use prior models based on allowable deformation of a template shape [66]. In addition, for tissue level case, the AW perimeter obtained after Lugol's solutions was assessed by examining the topography of the perimeter lines cut across the image contour with lines positioned in radial direction [27, 63, 79, 80].

Several techniques are applied to extract the size and shape features:

 (i) thresholding technique [60, 62, 77, 81];
(ii) clustering technique [70, 73];
(iii) fuzzy technique [69];
(iv) wavelet technique [82, 83];
(v) statistic techniques [13, 22, 78, 84].

At cellular-level, the size and shape features in cytology images are extracted using thresholding [62], clustering [70, 73], fuzzy [69], and wavelet techniques [83]. In the FISH images, the features are extracted using thresholding [60, 77, 81]. Besides, in the electromagnetic spectra, the features are extracted using statistical techniques [13, 22, 78, 84] and wavelet technique [82]. At tissue-level, perimeters were analyzed in terms of their topology changes such as perimeter' peaks [80]; van Raad et al. [68] used landmark technique of the closed contours to extract the perimeter features which differentiate normal and abnormal cervix. In the HSDI image case, the perimeter feature is extracted using landmark technique after an enhancement process [27]. Automated landmark extraction, including the extraction of the cervix boundary, detection of the Os (one of the anatomic region), and detections (and elimination) of specular reflections are used by [63, 79, 85].

#### 4.2.2. Topology Features

The topological features provide information on the structure of a tissue by quantifying the spatial distributionof its cells. For that, this approach encodes the spatial interdependency of the cells prior to the feature extraction. The features are applied for cellular-level case. The specific features implemented for cytology images are distribution of cell [72] and distribution of the nucleus [72], while the distances between the same color spots [60, 77], the distance between the centers of the two spots [60, 77], the gravity center of each colored spot [60], and the total number of red spots and green spots [60, 76, 77] have been implemented in the FISH images. The thresholding techniques are applied to extract the features in the cellular-level case [60, 72].

#### 4.2.3. Textural Features

Texture is a connected set of pixels that occurs repeatedly in an image. It provides information about the variation in the intensity of a surface by quantifying properties such as smoothness, coarseness, and regularity. At the cellular level, the existence of multinuclear cells [72] and the existence halos in cells [72] are used as features in the cytology image. Meanwhile, at the tissue level, the texture features are extracted from the AW region of the cervix image [61, 71, 86–91]. The texture is formed after giving the acetic acid or Lugol's iodine to the cervix surface as a sign of the abnormality.

There are several techniques applied for extracting the textural features in cervix images as follows.

- Wavelet technique [89].
- Mathematical morphological operations [71, 90].
- Clustering technique [86].
- Thresholding technique [62, 88].

At the cervix image of tissue-level, van Raad [89] demonstrated Gabor wavelet for extracting the textural features which outline the area of metaplastic changes, known as the transformation zone (TZ). The performances of the Gabor wavelet scheme achieve close to 80% accuracy in discrimination on the ROI. On the other hand, textural features (i.e., mosaic pattern) within the AW region are obtained from skeletonized vascular structures uniquely. The skeletonized vascular structures represented typical vascularity embedded in the normal and abnormal regions extracted by a series of mathematical morphological operations [71]. The series of mathematical morphological operations are gray-scale method, top hat transform, morphological opening with a rotating structuring element (ROSE), thresholding, and skeletonizing. Similarly, the textural features are extracted based on iterative morphological operations with various sizes of structural elements, in combination with adaptive thresholding [90]. Furthermore, combination of mathematical morphology and clustering based on Gaussian mixture model (GMM) is proposed to extract the textural features in the cervix image [86]. The algorithms are used to segment macro regions of the textural cervix images. Thresholding technique is used to segment tissues and nucleus as the texture for each application, respectively [62, 88, 92].

#### 4.2.4. Color Intensity Based Features

The color intensity-based features are extracted from the gray-level or color histogram of the image. This type of features does not provide any information about the spatial distribution of the pixels. The intensity histogram in a cell is employed to define features. In the case of cellular level images, the difference of color intensity can be used as features for the cancerous cells [72, 73]. Cytology image has a relatively darker color intensity composition than normal cells. The distinguishable patterns can be analyzed using the corresponding image's color intensity histogram [83]. Meanwhile, another feature to differentiate the abnormality of cervix using FISH image is the average intensity of each colored spot [60]. At the case of tissue level images, the changes in color and intensity correlate closely with changes in tissue type, severity of cervical neoplasia, and vessel patterns [61, 67, 86, 91, 93–97].

Several techniques used for extracting the intensity features are as follows.

 (i) Clustering technique [67, 86, 95, 97].
(ii) Watershed technique [93, 94].
(iii) Statistical technique [96].

van Raad [67] used a clustering technique (i.e., GMM) based MAP algorithm probability model in cervical images to extract color information features belonging to each of the tissue types in the cervix, such as the cervical canal (CC), the transformation zone (TZ), the squamous epithelium (SE), and the artifact named specular reflection (SR). Besides, mean-shift clustering is used to extract color and texture features of a tissue type [95]. Clustering based on the GMM is used in a joint color and geometric feature space to segment macro regions [86]. Similarly, [97] used a clustering technique (i.e., *K*-means clustering (KMC)) to generate an anatomical feature map for each cervical tissue type. The tissue regions defined by the anatomical feature map are further clustered into subregions. Watershed technique is used for a specific focus on the detection of lesion regions in uterine cervix images [93, 94]. Meanwhile, the spatial change of the AW lesion is extracted using color and texture information based on an opacity index that indicates the grades of temporal change [96].

As presented in Figure 4, possibility of the ratio and texture features can be extracted from FISH image for future works. As listed in Table 3, the features of the FISH image are area and radius for each colored spot. The ratio of the area for one colored spot and other colored spot can be possibly extracted. The ratio of the radius of one colored spot and other colored spots can be also possibly extracted. The texture of one FISH image integrally can be also extracted to differentiate the abnormality of the images.

### 4.3. Features Selection

After all the possible features for classification had been extracted, the selection of significant or dominant features can be conducted. Besides feature's extraction systems, the classification performance also depends on the selected features and the classification technique used. Feature selection is an important stage in classification, especially if it involves a large dimension of input features. By applying this feature selection stage, the original high dimensional inputs could be transformed and reduced into new lower dimensional features [98].

Generally, all possible extracted features can be used as the inputs for a classification system. However, irrelevant or noisy features could deteriorate between classes and increase the overlap in a non-linear manner. The noisy features can mix up the boundaries for the generalization performance of the classification system [99]. A clasifier with fewer inputs needs fewer weights to be adjusted, leading to better generalization and faster training [100]. Adding newer features can significantly lead to a reduction in the performance of the classification system [100].

Many researchers in computer vision based spectroscopy data applied the features selection techniques for cervical cells and other cell features [13, 51, 58, 84, 98, 101–110]. Generally, good performances in classification are achieved after applying the features selection techniques. Since the spectral data is heavily redundant, the selection of the significant wavelength as features in this case is vital.

For the image processing application, discriminant analysis (DA) and principle component analysis (PCA) are methods commonly used to find a linear combination of features which characterizes or separates two or more classes of objects or events. The resulting combination may be used as a linear classifier or, more commonly, for dimensionality reduction before later classification [111].

The DA works by creating a new variable called the discriminant function score which is used to predict to which group a case belongs. The discriminant function scores are computed similarly to factor scores (i.e., using eigenvalues). The computations find the coefficients for the independent variables (features) that maximize the measure of distance between the groups defined by the dependent variable. The disadvantages of the DA are the distribution of distance matrices in the same class to be singular if the dimension of the data is much higher than the number of training samples [112].

Besides, the PCA is mathematically defined as an orthogonal linear transformation that transforms the data to a new coordinate system. In the PCA, the greatest variance by any projection of the data comes to lie on the first coordinate (called the first principal component), the second greatest variance on the second coordinates, and so on. The first principal component corresponds to a line that passes through the multidimensional mean and minimizes the sum of squares of the point's distances from the line. The second principal component corresponds to the same concept after all correlations with the first principal component have been subtracted out from the points.

There are several researchers in cervical cancer application who use the PCA [13, 17, 113]. In their researches, the PCA is used as dimensionality reduction to improve the classification performance and decrease the training time of classifier. However, the disadvantages of the PCA consist in the fact that the directions maximizing variance do not always maximize information. In case, a great disadvantage of PCA is that it does not consider any class information [114]. This can lead to a loss of important discriminating information. In fact, the analysis showed that it was practically impossible to improve the classification error by this method [114]. Another disadvantage of the PCA is that it has high memory and computational requirements [115].

### 4.4. Classification

**Figure 5:**
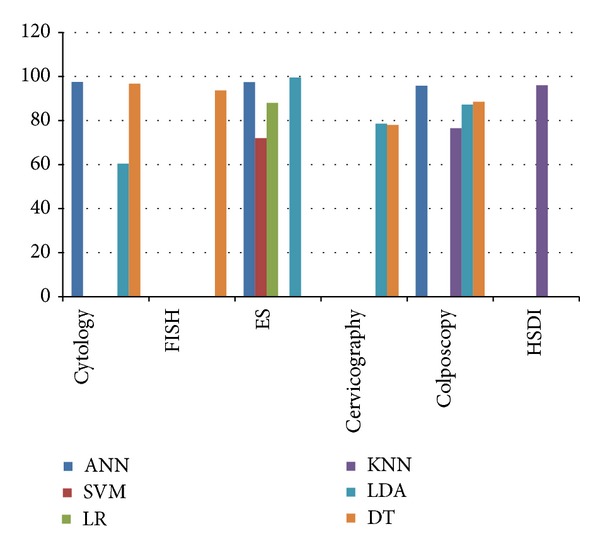
Performances of six classifiers generally for cervical precancerous data.

The effectiveness of the automatic cervical precancerous screening system is evaluated in this section. The classifiers mostly used for cervical cancer study in detail are artificial neural networks or neural network (NN) [56, 72, 73, 75, 78, 80, 82, 84], support vector machine (SVM) [116], logistic regression [12], *K*-nearest neighborhood (KNN) [80, 97, 117], linear discriminant analysis (LDA) [13, 22, 24, 61, 85, 88, 118, 119], and decision trees [56, 60, 94, 96, 120, 121], as listed in Table 4. The performances of the classifiers generally showed good results as presented in Figure 5.

Generally, each type of classifiers can be employed for all types of data. For example, the FISH or cervicography data can be classified using NN, SVM, logistic regression, KNN, LDA, and decision tree. However, it is important to know the advantages and disadvantages of the classifiers that might be considered as alternatives. Logistic regression is attractive for probability prediction, because it is mathematically constrained to produce probabilities in the range [0,1] and generally converges on parameter estimates relatively easily [122]. The disadvantages of the logistic regression are not designed to deal with high-dimensional data and cannot approximate any smooth polynomial function, regardless of the order of the polynomial or the number of interaction terms [122].

SVM's execution speed is very fast and there are no parameters to tune except the constant C. It is remarkably intolerant of the relative sizes of the number of training examples of the two classes. Since the technique is not directly trying to minimize the error rate, but trying to separate the patterns in high dimensional space, the result is that SVM is relatively insensitive to the relative numbers of each class. The possible disadvantages are large memory requirement [123] and the training time can be very large if there are large numbers of training examples [124].

Meanwhile, the NN architecture is initially not structured and the learning algorithm is responsible for the extraction of the regularities present in the data by finding a suitable set of synapses during the process of observation of the examples. Thus, NNs solve problems by self-learning and self-organization [125]. However, the neural network required long training time, and the results depend on the initialization parameters. It consisted of an arbitrary number of layers, and parameters [122]. Different combinations of number of hidden neurons, learning rate, momentum rate, activation function, epoch size, and initial weights have to be tried in order to produce better results [125].

Decision tree is relatively easy to interpret and to implement. Like SVMs and NNs, many methods for decision trees do not provide a probability of class membership, although some variants, in particular, classification and regression trees, do provide such probabilities. However, performance of all decision trees is dependent on both their method of construction and the amount of pruning (removal of highly specific nodes) performed [122].

KNN and LDA are methods implemented in numerous programs and easy to be implemented as classification tools. Both techniques have direct analytical solution and very good at detecting global phenomena (whereas decision tree detects local phenomena). However, they are simply defined and implemented, especially if there is insufficient data to adequately define sample means and covariance matrices. Both techniques only detect linear phenomena and are sensitive to individuals outside the norm.

From Table 4 and Figure 5, the NN results showed constantly higher performance results in terms of accuracy than the other classifiers. The result ranges are 78.7–99% of accuracy. Mostly, the accuracy results are higher than 90%. In detail, [56] achieved 78.7% of accuracy in their preliminary study in classifying more than 1000 data to be two classes. [72, 73, 75, 78, 80] successfully achieved more than 90% of accuracies (i.e., 99%, 97.5%, 91.4%, 95.8%, and 97.4%) to classify 400 data to be 2 classes, 550 data to be 3 classes, 78 data to be 5 classes, 283 data to be 2 classes, and 780 data to 3 classes, respectively.

As presented in Table 4, six types of data are used for classification purpose. All data have good capability to be used as intelligent classification data. The classification performances of the data are spread from range 60 to 99% of accuracies. Overall performance shows that cytology features and the electromagnetic spectra features give the higher accuracy than the other data. Many of the researchers that use the data gain accuracy values more than 90% such as performances using cytology data: 96.7% [120], 99% [72], 97.5% [73], and 91.4% [75] and performances using electromagnetic spectra data: 96.4% [22], 99.5% [13], 90% [24], 97.6% [119], and 97.4% [78]. Only few have performance less than 90% of accuracy.

The cytology combined with neural network gives the accuracy of up to 99% of accuracy to classify 400 data to be 2 classes, followed by neural network using the electromagnetic spectra features at 97.4% for classifying 780 data to be 3 classes. Greatly, the electromagnetic spectra features could achieve the higher accuracy only using discriminant analysis at 99.5% of accuracy. Therefore, based on Table 4, the better cervix data used for the automated diagnosis are the cytology and the electromagnetic spectra features and the best classifier used for the automated diagnosis system is neural network.

As reviewed, the intelligent classification system for cervical precancerous cells has been attempted and developed using two types of input attributes; cervical cell/tissue images and cervical cell spectra. Therefore, the systems have employed image and signal processing techniques for extracting features as the input attributes, respectively. Both systems could classify the cervical precancerous cells with high performances. The applications of image and spectra processing and classifier for cervical precancerous classification have been developed by many researchers in the world. The screening techniques have been proven to have better performance than the manual techniques. Thus, the intelligent classification system for cervical precancerous using the image and/or optical spectra as input is believed to have better classification performance and could be used as a second opinion to pathologists.

## 5. Summary

Six types of cervical precancerous data (i.e., cytology, FISH, electromagnetic spectra, cervicography, colposcopy, and HSDI) generally can be used for the intelligent screening of cervical cancer. Computer screening system for cervical cancer based on cellular level data, namely, cytology, FISH, and electromagnetic spectroscopy, achieved better results as compared to tissue level data such as cervicography and colposcopy.

Classification tools (i.e., ANN, SVM, logistic regression, KNN, LDA, and decision tree) generally can achieve good performances to classify the cervical precancerous data. The screening systems based on neural network technique are frequently applied due to the better results and potential of the technique to build a real time system.

The long training time of the neural network can be reduced by using the features selection stage in the computer screening system. The dimensionality reduction popularly done by using discriminant analysis and principal component analysis can be developed using new techniques that can be proposed as future work in this research field. The developed techniques will reduce the training time and improve the classification result of the neural network.

## Figures and Tables

**Table 1:** Comparison of the ability of the manual cervical screening methods.

Highlighted features	Cellular level				Tissue level			
	Pap smear	LBC	HPV-DNA	EMS	VILI/VIA	Cervicography	Colposcopy	HSDI
Low cost	V*	V*	V	V*	V	V	V*	V*
Short time	X	X	X	X	V	V	V	V
Not Subjective	X	X	V	V	X	X	X	X
Possible in real time	V	V	X	V	X	V	V	V

**Table 2:** Information about cervical screening instruments.

Information	PAPNET	AutoPap 300	FocalPoint	TIS
Input data	Pap smear only	Pap smear only	Pap smear and ThinPrep	ThinPrep only
Characteristic	Semiautomatic system	Automatic system	Automatic system	Automatic system
USFDA approval	Secondary screening	Primary screening	Primary screening	Primary screening

**Table 3:** The list of features that are extracted by different data.

	Cellular-level based features			Tissue-level based features		
	Cytology	FISH	Electromagnetic spectra	Cervicography	Colposcopy	HSDI image
Size	(i) Area of Cell [72], (ii) Area of Nucleus [72, 73, 75] (iii) Area of Cytoplasm [75].	(i) Area for each coloured spot [60, 76, 77]. (ii) Radius of each coloured spot [60],	Shift of peak frequency [24]	Perimeter of anatomical features [63, 79]	Perimeter of anatomical features [80]	Perimeter of acetowhite [27]

Shape	(i) Circularity of cytoplasm [75] (ii) Circularity of nucleus [24, 75]	Circularity of each coloured spot [60, 77].		(i) Circularity of cervix [66] (ii) Circularity or elliptical shape of Os region [66]		

Ratio	(i) Percentage of cell coverage [72] (ii) Ratio of nucleus to cytoplasm size [72, 75] (iii) Percentage of empty cells [72].		(i) Ratio of peak intensities [24, 78] (ii) Ratio of area under peaks [78]			

Topology	(i) Distribution of cell [72] (ii) Distribution of nucleus [72],	(i) Distances between the same color spots [60, 77]. (ii) Distance between the centers of the two spots [60, 77]. (iii) Center of gravity for each coloured spot [60], (iv) Number of red and green spots [60, 76, 77].				

Texture	(i) Multinucleus cells [72], (ii) Halos in cells [72].			Acetowhite region [86–88],	Acetowhite region [61, 71, 89–91]	

Color intensity	(i) Cell [72, 83] (ii) Nucleus [73] (iii) Cytoplasm [73]	Intensity of each coloured spot [60].		Anatomical features [86, 93, 94]	Anatomical features [61, 67, 91, 95–97].	

**Table 4:** The list of classifiers that are used by different studies.

	Cellular-level based features			Tissue-level based features		
	Cytology	FISH	Electromagnetic spectra	Cervicography	Colposcopy	HSDI image
Artificial Neural network	(3/1241/10/78.7) [56], (2/400/10/99) [72], (3/550/4/97.5) [73], (5/78/5/91.4) [75].		(2/361/13/74.4) [84], (2/201/3/87) [82], (3/780/22/97.4) [78].		(2/283/7/95.8) [80].	

Support vector machine			(3/63/10/72) [116]			

Logistic regression			(4/145/—/88) [12]			

*K*-nearest neighbors					(2/283/7/68.9) [80], (2/48/10/76.5) [97].	(7/371/5/95.96) [117]

Linear discriminant analysis	(5/230/15/60.4) [85].		(2/324/—/78) [118], (2/275/8/96.4) [22], (2/150/5/99.5) [13], (4/800/7/90) [24], (2/92/3/97.6) [119]	(2/100/—/78.5) [88].	(2/40/4/87.2) [61].	

Decision trees	(3/1241/10/77) [56], (—/61/2/96.7) [120].	(2/325/—/93.6) [60]		(2/211/—/78) [94].	(2/29/—/86) [121], (2/99/—/88.5) [96].	

The values given in bracket are number of classes/number of  data/number of features used/accuracy.
